# BioModels Database: An enhanced, curated and annotated resource for published quantitative kinetic models

**DOI:** 10.1186/1752-0509-4-92

**Published:** 2010-06-29

**Authors:** Chen Li, Marco Donizelli, Nicolas Rodriguez, Harish Dharuri, Lukas Endler, Vijayalakshmi Chelliah, Lu Li, Enuo He, Arnaud Henry, Melanie I Stefan, Jacky L Snoep, Michael Hucka, Nicolas Le Novère, Camille Laibe

**Affiliations:** 1 European Bioinformatics Institute, Wellcome Trust Genome Campus, Hinxton, CB10 1SD, UK; 2 Division of Engineering and Applied Science, California Institute of Technology, Pasadena, CA 91125, USA; 3 Department of Biochemistry, Stellenbosch University, Private Bag X1, Matieland 7602, South Africa

## Abstract

**Background:**

Quantitative models of biochemical and cellular systems are used to answer a variety of questions in the biological sciences. The number of published quantitative models is growing steadily thanks to increasing interest in the use of models as well as the development of improved software systems and the availability of better, cheaper computer hardware. To maximise the benefits of this growing body of models, the field needs centralised model repositories that will encourage, facilitate and promote model dissemination and reuse. Ideally, the models stored in these repositories should be extensively tested and encoded in community-supported and standardised formats. In addition, the models and their components should be cross-referenced with other resources in order to allow their unambiguous identification.

**Description:**

BioModels Database http://www.ebi.ac.uk/biomodels/ is aimed at addressing exactly these needs. It is a freely-accessible online resource for storing, viewing, retrieving, and analysing published, peer-reviewed quantitative models of biochemical and cellular systems. The structure and behaviour of each simulation model distributed by BioModels Database are thoroughly checked; in addition, model elements are annotated with terms from controlled vocabularies as well as linked to relevant data resources. Models can be examined online or downloaded in various formats. Reaction network diagrams generated from the models are also available in several formats. BioModels Database also provides features such as online simulation and the extraction of components from large scale models into smaller submodels. Finally, the system provides a range of web services that external software systems can use to access up-to-date data from the database.

**Conclusions:**

BioModels Database has become a recognised reference resource for systems biology. It is being used by the community in a variety of ways; for example, it is used to benchmark different simulation systems, and to study the clustering of models based upon their annotations. Model deposition to the database today is advised by several publishers of scientific journals. The models in BioModels Database are freely distributed and reusable; the underlying software infrastructure is also available from SourceForge https://sourceforge.net/projects/biomodels/ under the GNU General Public License.

## Background

Advances in molecular and cellular biology over the past few decades have triggered tremendous growth in available experimental data. To generate novel or insightful hypotheses from this enormous quantity of data is a significant challenge. Computational modelling can help meet this challenge by contributing to a deeper understanding of relevant chemical and biological phenomena based on their underlying mechanisms. Simulations of models can help investigate a complete biological process instead of considering smaller segments or aspects, detail a segment of a process or simplify a very large one, suggest or even direct future experiments, and predict the behaviour of a system under given conditions. Supporting these goals requires precise models that accurately represent biological systems in a quantitative manner.

To construct a large-scale comprehensive view of biological systems, several smaller models may need to be integrated. This can be difficult to accomplish, since models can exhibit significant variations even when purporting to cover the same domain space. They can come from different modellers, developed at different times from different perspectives, and be encoded in different formats. Consequently, some models cannot be practically reused, or even worse, may be entirely lost due to a lack of the necessary information that would allow them to be exchanged or converted.

The definition and adoption of standard and machine-readable formats for encoding quantitative models has already been recognised as a crucial first step for efficient exchange and reuse. CellML [1] and NeuroML [2] are such examples, but the Systems Biology Markup Language (SBML) [3], being adopted by more than 180 software systems ranging from simulators to model editors and databases [4], has so far been the most successful standard model exchange format in this field.

The next stage of infrastructure development for computational modelling is the creation of public repositories where models can be freely deposited and distributed in standardised formats. Models in these repositories should be curated according to agreed-upon standards, and annotated using community-developed controlled vocabularies, for instance with Gene Ontology [5,6] and Taxonomy [7,8]. Linking the components to external data resources, such as protein sequences from UniProt [9] or pathways from Reactome [10], can allow the unambiguous identification of the components. This in turn can enable members of the biomedical and life science communities to search and retrieve models, or parts of models, relevant to their research topics, whether that topic is a disease, a biological process, a given molecular complex, or something else.

We developed BioModels Database [11-13] precisely with these goals in mind, while other resources, such as ModelDB [14], JWS Online [15] or the CellML Model Repository [16,17], focus on different aspects. The resource is part of the BioModels.net initiative [18,19], which aims to (1) define community standards for model and simulation curation, (2) provide controlled vocabularies to define and link the terms used in systems biology, and (3) provide a free, centralised, publicly-accessible database for storing, searching and retrieving curated and annotated computational models. Here we describe the current structure of BioModels Database as well as its use.

## BioModels Database design and procedures

The BioModels Database server software uses a typical three-tier architecture, in which the data storage, processing and presentation are logically separated. The programming language used for the main development is Java [20], while some conversion-related processes (described below) are implemented using a combination of Extensible Stylesheet Language Transformations (XSLT) [21] and shell scripts.

### Interface

The web interface of BioModels Database is implemented using JavaServer Pages (JSP) [22]. Asynchronous JavaScript and XML (AJAX) [23] is used to make the web interface more dynamic and improve the responsiveness of many processes, especially in the model annotation interface where a given page does not have to be reloaded each time a curator makes an incremental annotation. AJAX also makes user-level features such as model component extraction more interactive.

### Authentication and User Roles

There are four main types of user roles defined in BioModels Database: The *Public user *role is assigned by default, and requires no registration. Public users can access the database to search, view and download models, as well as to run simulations. They can also submit models. The remaining roles of *Curator*, *Annotator *and *Administrator *are used by database curators and developers; they provide additional permissions beyond those of *Public user *and require specific registration.

### Model Storage

Quantitative information, kinetic laws and model entities are all stored in SBML files. Model metadata are stored separately in a MySQL database [24] and not in the SBML file. This simplifies the management of annotations, especially during the annotation phase when the data are updated frequently. Annotations are re-inserted into the SBML files during the release process, allowing users to directly download the fully annotated model files. Moreover, each model's history is tracked using Subversion [25].

An earlier version of BioModels Database stored model XML [26] files in Xindice [27], a native XML database. However, the increasing popularity of BioModels Database as well as an ever-greater number of models exceeding the file-size limit of Xindice forced us to redesign the system. The current version parses and builds indexes of model elements (e.g., name, identifier and notes) using Apache Lucene [28]. Queries based on these indexes are fast and efficient in terms of server memory and CPU usage.

### Database Schema

**Figure 1:**
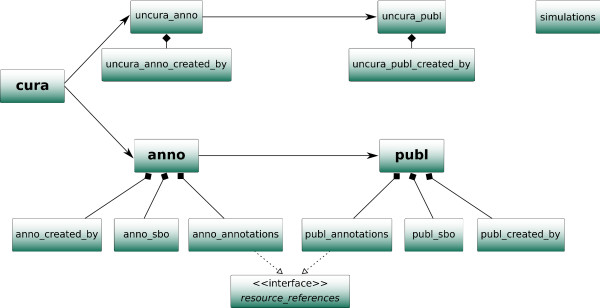
**Database structure**. Shown here is a schematic of the relational database structure used by BioModels Database. It depicts the different database tables and their relationships. The three main steps of the pipeline (curation, annotation and publication) are organized by the three main tables, cura, anno and publ, respectively. The two branches can be distinguished by the fact that the tables related to the non-curated branch are prefixed with uncura.

The metadata for all models (submission date, modification date, model format, authors' information, etc.), including references, are stored in a set of relational tables in the MySQL database (Figure 1). The design of these tables also reflects the different stages of the BioModels Database pipeline.

### Servers and Mirrors

BioModels Database runs on a server cluster configured with a failover mechanism. Faster access for North American users is provided through a mirror at the California Institute of Technology [29].

### Format converters

The converters from SBML to CellML [1] and to SciLab [30], as well as the converter from CellML to SBML, are written in XSLT [21]. The converters from SBML to XPP, BioPAX [31,32], Portable Network Graphics (PNG) [33,34] and Scalable Vector Graphics (SVG) [35] are written in Java. The Java converter from SBML to the Virtual Cell Markup Language (VCML) [36] is provided by the Virtual Cell team [37]. Converter details and source code are available online [38].

### Submodel generation

From the model overview interface, one can select species, reactions and/or compartments in order to generate a submodel containing these specific elements. This function relies on a Java library developed by the BioModels Database team. The library parses a model and extracts the submodel in a four-step procedure. Firstly, it extracts the species and compartments selected by the user, along with the reactions they are involved in; secondly, it fetches the selected reactions; thirdly, it retrieves the species and compartments involved in all previously obtained reactions; finally, it extracts the compartment types, species types, rules, events, parameters, units, and function definitions needed to build a model which is valid SBML.

### The BioModels Database pipeline

**Figure 2:**
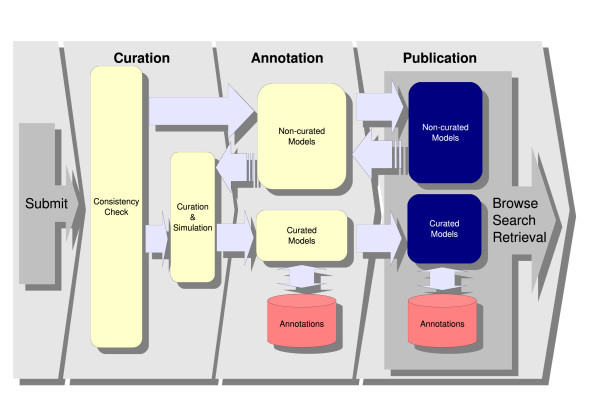
**BioModels Database pipeline**. The BioModels Database pipeline encompasses all the steps undergone by each model, from its submission to its publication. This figure illustrates the sequence of steps. It encompasses both public branches of the database (curated and non-curated) as well as the possibility of curating and annotating models already published in the non-curated branch.

The BioModels Database pipeline (Figure 2) manages all models from their submission to their publication. Models submitted to the database are not made publicly visible immediately upon submission; rather, they undergo a series of curation and annotation processes in order to ensure a consistent level of quality. As models pass through the pipeline, additional information is added to facilitate their reuse and the ability of software tools to perform functions such as searching, simulation, conversion or merging.

#### Model Submission

Model submission is open to the public. BioModels Database currently accepts models encoded in SBML as well as CellML format.

BioModels Database currently only distributes models published in peer-reviewed literature. During the submission process, submitters are required to provide an appropriate publication reference. This reference can be a PubMed Identifier (PMID) [39], a Digital Object Identifier (DOI) [40], or a URL. The publication reference helps other users to identify the model; it also improves the accuracy of database search engine. BioModels Database automatically searches for the reference in CiteXplore [41] and stores the retrieved data, including journal details, authorship, and abstract, in its internal indexes. Models not yet published will not have a publication reference; however, they can still be submitted to the database, and the reference data can be added later by the curators.

BioModels Database performs numerous consistency checks during the model submission procedure. The model must be syntactically valid XML, as well as valid with respect to its encoding schema. Errors detected during submission are reported to the submitter. A submission is successful only after all errors have been corrected. Following successful submission, a confirmation email notifies the submitter of the unique submission identifier assigned to the model. Each such identifier is composed of the character sequence "MODEL" followed by ten digits extracted from the timestamp of submission, and being unique and perennial, the identifier can be quoted in subsequent publications. A notification is also sent to the BioModels Database curator team to inform them that a new model has entered the curation pipeline.

We distinguish several "actors" in the process from model creation to its publication on BioModels Database:

• *The model's author *(*s*) is (are) the author(s) of the reference citation (i.e., the peer-reviewed article from which the model originates). Concerns regarding the biological basis of the model (e.g., the presence of an interaction not documented in the scientific literature, or behaviour differing from that expected of the biological process) should be directed to the model's author(s).

• The model's *encoder *(*s*) is (are) the person(s) who actually encoded the model in its present form. There may be several encoders, including the BioModels Database curators if they have to modify a model significantly. The encoder(s) should be contacted if there is a problem with the structure of the model (initial conditions, kinetics parameters, reaction scheme etc.).

• BioModels Database also defines the model *submitter *(*s*) as the person or persons who submitted the model to the repository. The submitter(s) should be contacted if there is a problem with the original model encoding or annotation.

Any specific concerns about a model can also be reported to the BioModels Database curators through an online form provided for this purpose on the website.

#### Model Curation

Successfully-submitted models are then queued into the curation pipeline, where several tasks are performed.

1. If a model is submitted in an old level or version of the SBML format, BioModels Database curators will convert it to the latest level and version of SBML, unless the curators believe that such conversion is likely to cause information loss or inaccuracies. Models submitted in CellML are converted to the latest SBML level and version since the BioModels Database software (annotation interface, simulation tool, etc.) is built around the SBML standard.

2. Further consistency checks are performed on the model using libSBML [42] and SBMLeditor [43]. This includes checks for identifier and unit consistency as well as for mathematical expression validity (more specifically, MathML [44] validity), among others.

3. Curators manually check that the encoded model faithfully represents the model described in the reference publication. This includes verifying the structure of the model, such as the relationships between variables and mathematical relationships, as well as the nomenclature used in the model components. It is important to emphasise the fact that, during this step, the structure of the submitted models may be modified by the encoders to reflect the structure of the model described in the paper.

4. Curators download the model and run simulation experiments under the conditions defined in the reference publication. These tasks are performed using several simulation tools, at least one of them being different from the tools used by the original authors of the model. (The latter requirement helps guard against software-specific behaviours or hidden dependencies.) The tools most commonly used are COPASI [45,46], the SBML ODESolver [47] or the facilities provided by the Systems Biology Workbench [48]. If the results cannot be reproduced, curators contact the model author(s), for clarification or discussion regarding any issues that have arisen. Once the results correspond to the paper, curators upload a typical results set to the database, together with comments on how, and with which tools, it was obtained.

5. The curators give the model a consistent and meaningful name following the general scheme *Author Year_Topic_Approach*. Examples include the names *Levchenko2000_MAPK_noScaffold *(referring to the model identified by the BioModels identifier "BIOMD0000000011") and *Edelstein1996_EPSP_AChEvent *(referring to the model "BIOMD0000000001").

After the curation phase, a model is moved into one of two branches depending on the outcome of curation as well as certain other criteria. In the *curated branch*, models are compliant with the MIRIAM (Minimum Information Required in the Annotation of Models) reporting guidelines [49]. MIRIAM compliance requires models to (1) be encoded in a public standard format, (2) be clearly related to a single reference, (3) correspond to the biological processes listed in the reference publication, and (4) produce the simulation results given in the reference publication using the same values and parameters. Models placed in the curation branch satisfy these requirements because, respectively, (1) each model is converted into SBML and validated, (2) each comes from a peer-reviewed published article, and each is verified by the curators to correspond to its reference description in both (3) structure and (4) results. By contrast, the *non-curated branch *is reserved for models that are valid SBML but either do not satisfy the full requirements for MIRIAM compliance, or have not been curated fully due to limited resources by the BioModels Database curation team. For example, non-kinetic models such as pathway and interaction maps, as well as steady-state analysis models, are stored in this branch because it is generally not possible to verify their results using simulations. Other models that are placed in the non-curated branch include spatial and boolean models that contain proprietary annotations needed for their interpretation, and models that do not reproduce the required results.

Once a model is moved to the curated branch, a new BioModels Database identifier is generated and assigned to it. This identifier is composed of the character sequence "BIOMD" followed by ten digits reflecting the model's position the branch, for example "BIOMD0000000216" for the 216^th ^model successfully curated. As is the case for submission identifiers, curation identifiers are unique and permanent, and will never be re-assigned to a different model, even if for some reason a particular model must be retracted from the database.

#### Model Annotation

MIRIAM compliance requires a model to have (1) a unique meaningful name, (2) a reference citation linking the model to a unique publication, (3) the name and contact information of the model author(s), (4) the date and time of model creation and last modification, and (5) a precise statement about the terms of distribution. This information is generally the first to be added to a model during the annotation phase.

In publications describing models, the different elements such as specific genes, proteins and metabolites, or the organisms from which the model is derived, are often described in the text or just given convenient or biologically non-meaningful names without any links to reference external resources. Furthermore, the names of elements in the models most often do not allow users to directly relate them to a precise biological function or physical entity. This can greatly diminish model interpretability by both users and software tools. Annotating model elements helps avoid these problems, allowing unambiguous identification through reference to appropriate external resources (using perennial URIs), such as terms from controlled vocabularies (Taxonomy, Gene Ontology, ChEBI ontology [50], Enzyme Nomenclature [51], etc.) and links to other databases (UniProt, KEGG [52], Reactome, etc.). In order to enhance the semantics of models, terms from the Systems Biology Ontology (SBO) [53] may also be added in the annotation phase. Theoretically, all resources listed in MIRIAM Resources [54] could be used for annotating model elements. Annotations are used to improve the accuracy of search procedures, as well as provide additional information about the model components. They can also be useful in users' analyses of the models, for instance in clustering [55] or merging [56] procedures.

Annotating each model component with the most relevant resource terms requires great efforts, especially since the number of submitted models has grown rapidly. The 17^th ^release of BioModels Database (April 27^th ^2010), contains 18,950 cross-references (links to external resources contained in the annotations). This is a modest number when compared to the total number of species (37,852) and reactions (44,886) involved in the existing 473 models. This mostly reflects the lack of annotation in the non-curated branch, which is mainly due to limited curator resources (in terms of both time and specific knowledge about the models). Low annotation is also sometimes caused by a lack of adequate or suitable resources, as in the case of molecular entities that exist in a model only for simulation purposes. Moreover, biological data resources are often slightly lagging behind newly generated knowledge, and it is possible that a particular resource does not offer the relevant information at the time the model is annotated. In the case of hierarchical controlled vocabularies, such as Gene Ontology or ChEBI, there is the option to use a term at a higher level of abstraction if the required precise term does not currently exist. Most often, curators nevertheless find ways of adding some information, even if not in an optimal fashion. Model annotation needs to be, and indeed is, a continuous process.

#### Model Publication

Following the curation and annotation phases, the final stage in the model processing pipeline is model publication. The model is tagged as ready for publication, and becomes publicly available online with the next release of the BioModels Database. New releases of the database are issued two to four times per year.

## Description

The large range of features provided by BioModels Database allow users to quickly locate models of relevance for them, analyse them (and understand their structures), simulate them, extract submodels, or download them in various formats (whether text-based or graphical). These facilities are available via a web browser, or can be directly accessed from other tools by using the accompanying web services.

### Model Browsing

The most basic way of finding a particular model is to identify it from the list of available models. Links to the lists of curated models and non-curated models can be found on the homepage of BioModels Database; the same links are also available from the menu at the top of each page of the site. The lists are presented as tables whose columns display several different model characteristics; within these tabulated views, a user can sort the list of models by *model identifier*, *model name*, *publication identifier *or *the date of last modification*, by clicking the appropriate column heading.

**Figure 3:**
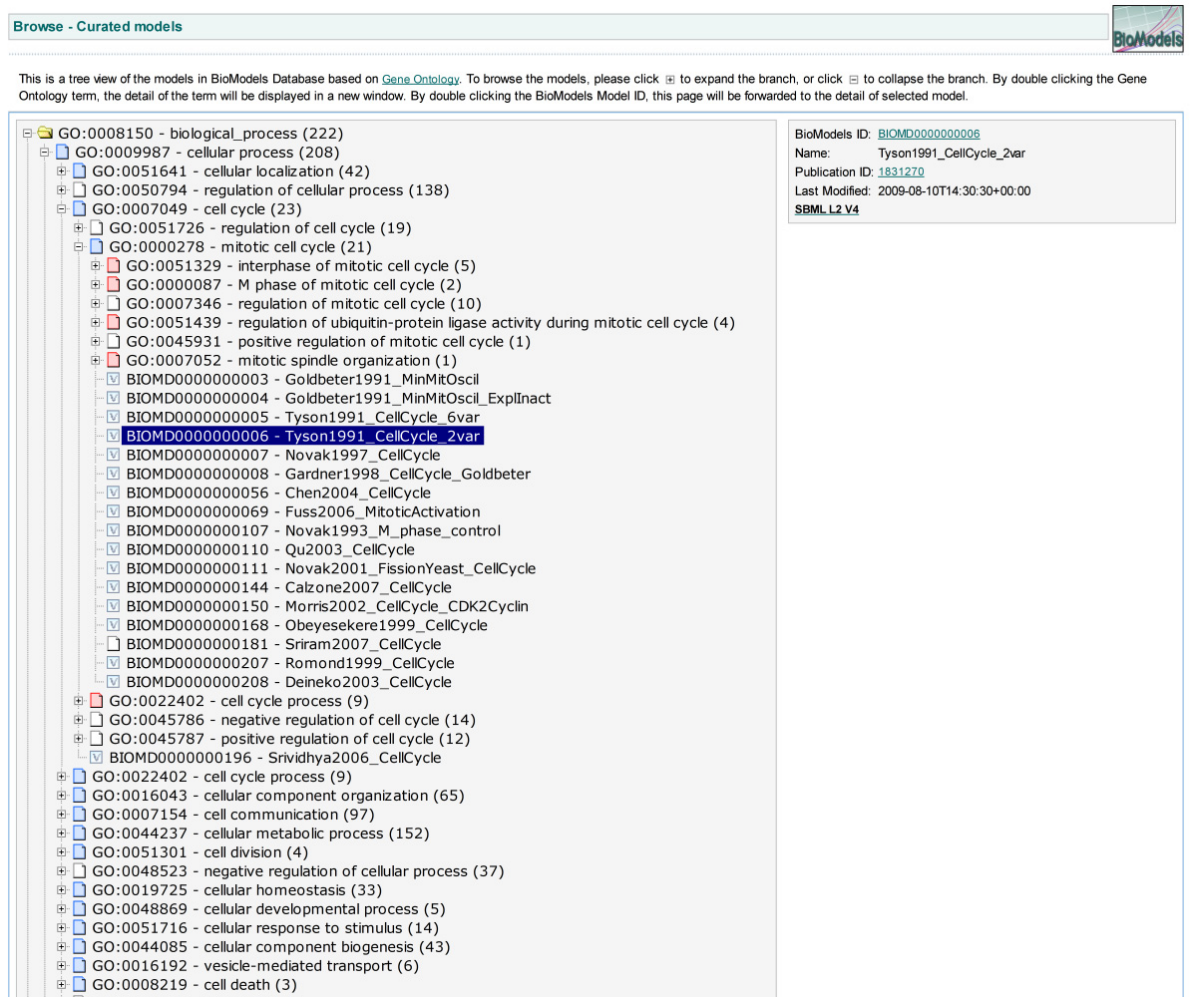
**Models tree based on Gene Ontology**. BioModels Database provides users with three primary facilities for finding and discovering models: the system's search interface, the browsable list of all models, and an alternative list based on Gene Ontology (GO) terms. A screen image of the last alternative is shown here. The GO-based view is derived from the annotations of models in the database; the annotations of all models are collected and used to generate a pruned GO tree, and this tree can be browsed in order to find models annotated with a specific GO term.

**Figure 4:**
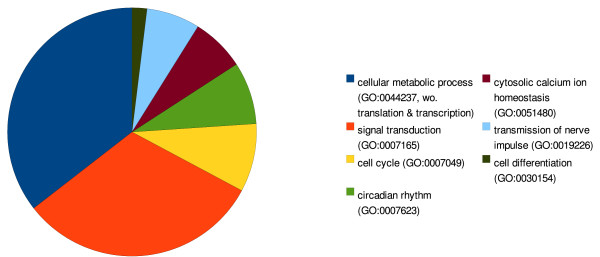
**Thematic content of models**. Categorisation of models in BioModels Database using the Gene Ontology (GO) terms present in each model's annotations. This chart was generated by enumerating models in the database whose annotations refer to children of the GO terms listed here, after first removing certain GO terms (*translation*, GO:0006350; *transcription*, GO:0006412; and *cellular metabolic process*, GO:0044237) that appear across different categories, and hence would have biased the analysis.

An alternative to simply browsing the lists of models is available in the form of a tree-structured browser based on the Gene Ontology (GO) terms used in the annotation of models in the database. A navigable, pruned, subtree of GO is automatically generated by the system, allowing users to explore the database thematically. The parenthesised number that appears next to each branch of the GO tree indicates how many models within that branch contain that particular GO term. Expanding the GO tree branch allows a user to drill down to child terms and find models annotated with those more specific GO terms (Figure 3). The extensive GO term coverage within BioModels Database is illustrated in Figure 4.

### Search and Retrieval

**Figure 5:**
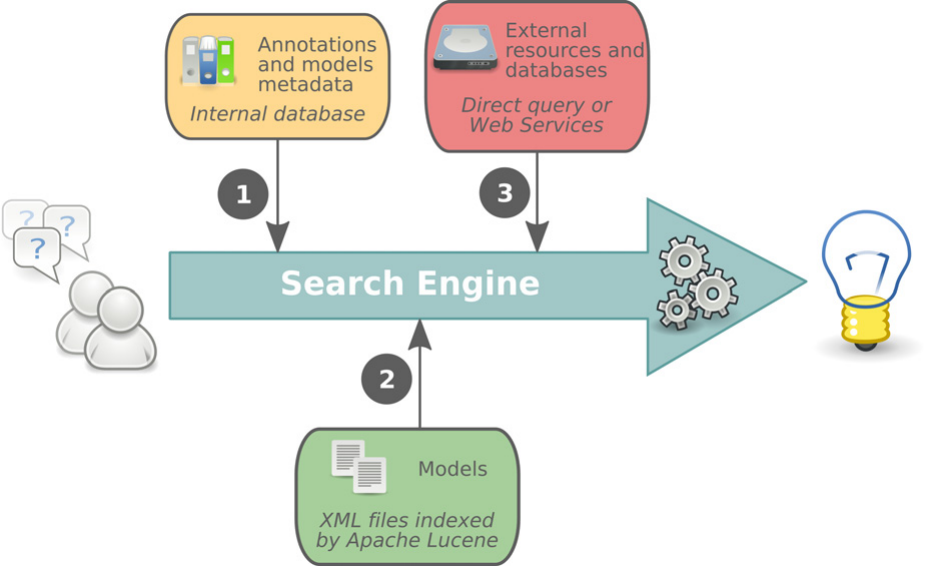
**Search engine**. The BioModels Database search engine processes three different types of data in order to provide an accurate result. First, it searches the annotations (by querying the internal database), then the models (using Lucene), and finally, data linked from external resources. Searching for the last is accomplished via direct connection to remote databases and by using remote web services. Ultimately, all the results are collected, processed to remove duplicates, then classified based on which branch the models come from, ordered, and finally, returned to the user.

BioModels Database incorporates a powerful search engine that allows users to quickly locate models of interest. In order to find relevant models, the algorithm performs several searches based on different data, then performs an inclusive disjunction (OR) to combine the results (Figure 5). The searches are performed sequentially as follows: (1) querying metadata, publications and annotations, (2) searching the model bodies, and (3) searching supplementary information from external resource databases. More specifically:

1. The search begins with the metadata (annotations) of all models in the database. Model metadata is used to facilitate the understanding, characteristics, and management of the model. It may consist of its name, identifier, timestamp, comments from curator(s), etc. The annotations of models include publication information, authors, terms from controlled vocabularies, and links to external resources. Metadata and annotation are supposed to best reflect the nature of a model, since they represent a verified mixture of curator input and algorithmic import.

2. The next step consists of searching through the SBML files of the models. For example, the 'notes' fields are examined, as they usually contain some information describing the model elements to which they are attached.

3. Finally, because it is impractical for BioModels Database to duplicate and keep up-to-date all relevant information available from model cross-references, several external databases are searched on demand through direct connection or using web services. During this step, the search engine checks available supplementary information such as synonyms and detailed synopsis.

**Figure 6:**
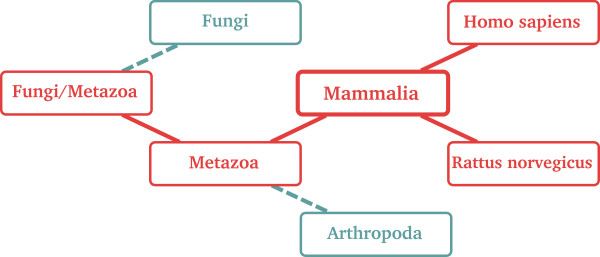
**Taxonomic search**. When a user's search is based on a taxonomic term, the BioModels Database search algorithm considers the entire taxonomic hierarchy. For example, searching for the term "mammalia" will catch not only models annotated with the term *Mammalia*, but also models annotated with terms related to it, such as *Metazoa*, *Homo sapiens *or *Rattus norvegicus *(represented in red in this figure).

The system performs some post-processing of the search output in order to deliver better results for user consumption. For example, when the user performs a search using a taxonomic term, the engine traces the whole hierarchy in order to find related models. This means that a search based on the term *mammalia *will return not only models associated with *mammalia*, but also models annotated with its descendants and ancestors (Figure 6). The logic of this is that a model describing, say, a system of *Homo sapiens*, or of *Rattus norvegicus*, is a model describing a system of *mammalia*. Similarly, a model that is valid for all *metazoa *or all *vertebrata *will be valid for *mammalia *too.

Models can also be retrieved directly by using either of the two permanent and unique identifiers assigned to the model: the submission identifier, and the curation identifier.

### Model Presentation

**Figure 7:**
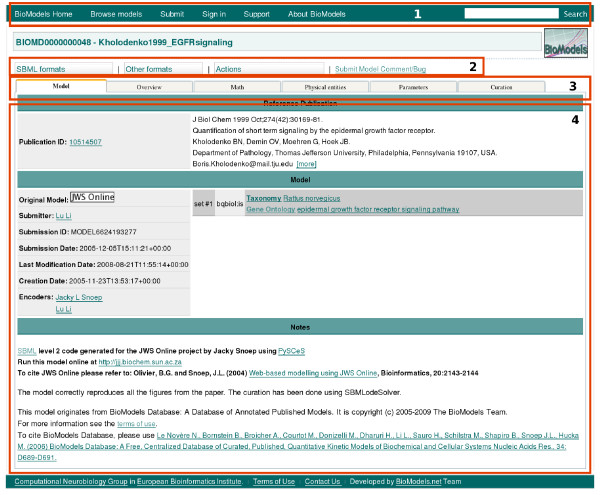
**View of a model page**. This screen image shows the interface of BioModels Database as it displays the model Kholodenko1999 EGFRsignaling (BIOMD0000000048). As illustrated here, the display of a model in the system is divided into several areas. The areas have been highlighted and numbered here for discussion purposes. The first area, across the top, contains general links for accessing the various features of BioModels Database. The second allows the user to perform actions specific to the model currently displayed (for example, to download the model in various formats or simulate it online). The third area contains all the different views of the model in separate tabbed window panes; each tabbed area is dedicated to a given aspect of the model (e.g., overview, mathematics, model entities, parameters, curation information, etc.). The fourth area is used to display content specific to the currently selected view of the model.

The model presentation page provides access to all of the information stored about a given model, as well as all the system actions available to the user (Figure 7). Elements are hyperlinked between the different views in the presentation of the model. In addition, each annotation is hyperlinked to detailed information about the annotated entity. When an annotation links to an external data resource, the contents of the linked-to resource entry are displayed in a new window in the user's web browser.

Within the model presentation page for a given model, the detailed description is separated into categories organised into a set of six corresponding tabs (area 3 in Figure 7):

• The *Model *tab displays general information about the model and its creation. The uppermost region of the tab summarizes the peer-reviewed, published article that describes the model. In the region below the publication information, a link provides access to the file originally submitted, as well as information about the encoders and the dates and times of model creation and last modification. Annotations displayed with the model refer to the model as a whole and indicate such things as the biological processes being modelled or the taxonomic coverage of the model.

• The *Overview *tab provides quick access to all the model components, that is, the mathematical relationships, physical entities, parameters and other elements comprising the model. Users can select components of interest, and that selection is subsequently reflected when they view the other tabbed panels. Clicking 'Create a submodel with selected elements' generates a model subset containing the selected components and all the components necessary to build a valid SBML model. This submodel is displayed in a new tab, where a link is available to allow the user to download it.

• The *Math *tab lists all of the mathematical constructs used to describe the relationships and the time evolution of the model's variables. These constructs include reactions, events, and explicit mathematical formulae (SBML rules). Each construct is accompanied by a rendering of the mathematical equation, as well as relevant hyperlinked annotations.

• The *Physical entities *tab lists the spatiotemporal entities (i.e., compartments and entity pools) contained in the model, along with their initial quantities and relevant annotations.

• The *Parameters *tab lists all parameters used in mathematical expressions. Parameters whose scope is limited to a reaction are grouped together. Parameters whose values are determined by mathematical expressions are linked to the relevant portion of the *Math *tab.

• The *Curation *tab displays representative curation results, obtained by the curators by simulating the model under the conditions defined in the reference publication. This tab includes graphical plots and comments from the curator.

### Export Formats

The *SBML formats *menu (area 2 in Figure 7) allows a user to download the model in various versions of SBML [3]. The version used to produce the curation figures is emphasised to indicate it is the only one tested by the curators. The other SBML versions are generated by an automatic conversion process.

The *Other formats *menu provides access to other (non-SBML) model representation formats, such as CellML [1], BioPAX [31,32], and the Virtual Cell Markup Language (VCML) [36]. To permit a given model to be simulated conveniently, BioModels Database also provides downloadable configuration files for open tools such as XPPAUT [57,58] and SciLab [30]. Finally, a human-readable report in the Portable Document Format (PDF), produced using SBML2LaTeX tool [59], is also available from the same menu.

The *Actions *menu provides access to graphical representations of the model's reaction networks, in the form of both static (PNG -Portable Network Graphics- and SVG -Scalable Vector Graphics-) as well as dynamic (interactive Java applet) presentations. A utility to convert graphs into the Systems Biology Graphical Notation (SBGN) [60] is currently being developed. The *Actions *menu also provides access to the online simulation tools, described below.

### Model Simulation

BioModels Database embeds SOSlib [47] to provide a basic online simulation tool. A given model can be simulated using this facility by selecting the 'BioModels Online Simulation' item from the *Actions *menu (area 2 in Figure 7). Once the user selects the species to be displayed and the duration for which the simulation should be performed, the simulation task is submitted to a computing cluster on the server side. The results of the simulation are returned in both graphical and textual form. For many models, an additional and more flexible simulation tool is available thanks to a collaboration between BioModels Database and JWS Online [61]. The JWS Online simulation system is available from the *Actions *menu.

### Model of the Month

Every month, a modeller picks a model of his/her choice and writes a short article that elaborates on the model. The article places the model in its biological and theoretical background and discusses its structure and the results of its simulation. This article is then published on the BioModels Database website as a *Model of the Month *[62]. Such articles make selected models more easily accessible to beginners, and may help them understand their context and significance.

### Web services

BioModels Database provides web services with a range of features to enable other software to programmatically search and retrieve up-to-date models and their associated data, and to extract submodels [63]. For example, tools such as the Virtual Cell [36], CellDesigner [64] or the Systems Biology Workbench [48] use these services to provide their users direct access (from within their tool) to hundreds of models. The services available are defined in a Web Services Description Language (WSDL) [65] file that enables software to easily understand available functions and their usage. BioModels Database web services use the Simple Object Access Protocol (SOAP) [66] to encode requests and responses. This allows standardised communication through HTTP [67] without the hindrance caused by proxies and firewalls. The complete list of available methods, as well as a Java library and the associated documentation, are provided on the BioModels Database website [68].

## Discussion

**Figure 8:**
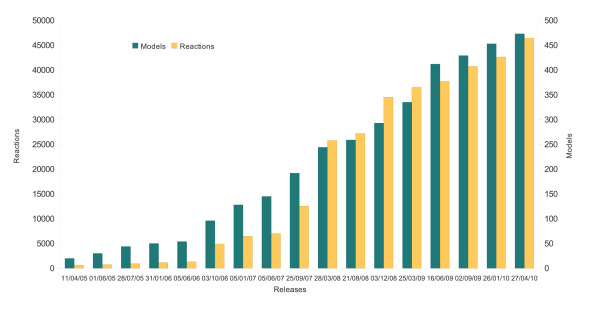
**Growth of BioModels Database**. Graph depicting the number of models (green) and the number of reactions (yellow) stored in BioModels Database at each release of the database made so far. The number of reactions includes SBML "rate rules", since some models only use rate rules. The graph illustrates that not only has the number of models increased approximately ten-fold since 2005, but the average complexity of those models has nearly tripled in the same period.

BioModels Database has become a recognised database in the computational systems biology field. It now contains an appreciable number of models, and indeed as far as we are aware, it is the largest public database of its kind today. On April 27, 2010, BioModels Database announced its 17^th ^Release, allowing freely available public access to 249 curated and 224 non-curated models. The number of models deposited in BioModels Database has nearly doubled on a yearly basis (Figure 8) since its inception in 2005. The number of reactions, species and annotations has increased even faster as a consequence of the fact that larger and more complex models are being submitted.

### Model sources

The models stored in BioModels Database come from several sources. Modellers themselves can submit their own models for inclusion in the database. In addition, many models are created from journal articles found in the literature by BioModels Database curators. Other models come from exchange with other collaborative model repositories, such as the former SBML model repository (Caltech, USA), JWS Online [61,69], the Database Of Quantitative Cellular Signaling (DOQCS) [70,71], and the CellML repository [16].

Several publishers of scientific journals recommend model submission to BioModels Database, including Nature Publishing Group, Public Library of Science, and BioMed Central. Following deposition, authors can quote the unique model identifier in their paper, allowing readers to download the model as soon as the paper is published. Some other journals, as part of their peer-review process, advise authors to deposit their computational models into other databases. JWS Online is used for this purpose by the journals Microbiology, FEBS Journal, IET Systems Biology, and Metabolomics. Those models are incorporated into BioModels Database after conversion from their native JWS Online format.

At present, BioModels Database focuses on storing models that can be encoded in SBML. Typically, these models represent activities, interactions or other dynamic phenomena in biochemical networks. BioModels Database also accepts other quantitative approaches such as steady-state models, and qualitative types of approaches, such as logical model; however, these other model types are mostly put into the non-curated branch, because a crucial part of the curation process involves verifying that a model reproduces the exact numerical results reported in the reference article describing the model, and we currently do not have processes for these other model types.

### Future development plans

We envision several improvements and additions to BioModels Database and its facilities. Planned developments include:

• Implementation of a versioning system to allow users the ability to retrieve and compare different revisions of a given model, including its annotations. This is a much needed feature, specially for efforts like the Minimum Information About A Simulation Experiment (MIASE), which aims at enabling the reproducible description of simulation experiments.

• Additional support for the submission of emerging or developing formats, such as the recently-released SBML Level 3 [72] and VCML [36].

• Improvements to the embedded search engine. One such improvement will be the introduction of a relevance ranking scheme for retrieved models, based on their annotations and data stored by external resources.

• Introduction of an annotation helper tool that will suggest appropriate annotations to the curator. Such a feature can incorporate tools such as semanticSBML [56], SAINT [73], or libAnnotationSBML [74].

• Distribution of more information with the models. We envision providing SED-ML [75] files in the future. This will allow users to download machine-readable descriptions of the simulation experiments realised during the course of the work that led to the publication of the model.

## Conclusions

Computational models are becoming ever more important in various aspects of the life sciences. This is reflected in the vast increases in both the number and the complexity of quantitative kinetic models in BioModels Database (Figure 8). This in turn necessitates the ability to reuse model components, and to build upon pre-existing models. BioModels Database was designed to address these needs.

BioModels Database is a freely available resource for storing curated and annotated versions of peer-reviewed, quantitative models of biological interest. Models are distributed in several forms, ranging from standard model file formats to graphical notations. Besides the analysis tools built into the web interface, BioModels Database offers a variety of useful features and tools to enable other software to programmatically search and retrieve models or submodels, construct large models from components, and access additional up-to-date information. Because the models stored in the database are thoroughly curated by humans, they can be used for teaching purposes, or to study specific biological processes. Moreover, since the models cover a wide range of domains, the whole set can be used for development and testing of simulation tools.

The BioModels Database pipeline, which encompasses the curation and annotation processes, ensures the correctness and quality of the models. The pipeline meticulously ensures syntactic correctness, logical model composition, the accurate capture of biological information, as well as confirmation that the model published will, within reasonable bounds, reproduce the behaviour attributed to it. Together with the cross-references that are embedded into each model, this provides the community with reliable and reusable models.

## Availability and licensing

All models stored in BioModels Database are freely accessible and reusable by all commercial and academic users. Once downloaded, modified models should be renamed and all author attributions removed prior to distribution of the modified model. This prevents the modified model being mistaken for the original present in the database, and should preclude any ensuing confusion this would cause. The full terms of use are accessible from a link at the bottom of every page on the BioModels Database website [76].

BioModels Database itself is an open-source project; the software is distributed under the GNU General Public License [77]. The database schema and code for both Web Application and Web Services are available from the BioModels SourceForge repository [78]. All converters are also available under the same license. This permits anyone to download and install a local version of the complete system, which may be useful for those who wish to store their own models privately or to integrate part or all of the system into their own software infrastructure.

## Authors' contributions

The work presented here was carried out by the authors in collaboration: CLi and MD, original developers of BioModels Database; LL and NR, converter and export developers; HD, LE, VC and EH, created, curated and annotated models; MIS, coordinator of the Model of the month; AH, developed the SBML to BioPAX converter; JLS, developed and maintained JWS Online; MH, provided coordination, SBML knowledge and grant support; NLN, curation, project instigation and coordination; CLaibe, feature development and current project coordinator. All authors have read and approved the final manuscript.
